# Validation of the diabetes, hypertension and hyperlipidemia (DHL) knowledge instrument in Malaysia

**DOI:** 10.1186/1471-2288-12-18

**Published:** 2012-02-24

**Authors:** Pauline SM Lai, Siew Siang Chua, Ching Hooi Tan, Siew Pheng Chan

**Affiliations:** 1 Department of Primary Care Medicine, Faculty of Medicine, University of Malaya, Kuala Lumpur, Malaysia; 2 Department of Pharmacy, Faculty of Medicine, University of Malaya, Kuala Lumpur, Malaysia; 3 Pharmacy Department, University Malaya Medical Centre, Kuala Lumpur, Malaysia; 4 Department of Medicine, Faculty of Medicine, University of Malaya, Kuala Lumpur, Malaysia

**Keywords:** Validation, Diabetes, Knowledge instrument, Malaysia

## Abstract

**Background:**

Patient's knowledge on diabetes, hypertension and hyperlipidaemia and its medications can be used as one of the outcome measures to assess the effectiveness of educational intervention. To date, no such instrument has been validated in Malaysia. Therefore, the aim of this study was to evaluate the validity and reliability of the Diabetes, Hypertension and Hyperlipidemia (DHL) knowledge instrument for assessing the knowledge of patients with type 2 diabetes in Malaysia.

**Methods:**

A 28-item instrument which comprised of 5 domains: diabetes, hypertension, hyperlipidemia, medications and general issues was designed and tested. One point was given for every correct answer, whilst zero was given for incorrect answers. Scores ranged from 0 to 28, which were then converted into percentage. This was administered to 77 patients with type 2 diabetes in a tertiary hospital, who were on medication(s) for diabetes and who could understand English (patient group), and to 40 pharmacists (professional group). The DHL knowledge instrument was administered again to the patient group after one month. Excluded were patients less than 18 years old.

**Results:**

Flesch reading ease was 60, which is satisfactory, while the mean difficulty factor(SD) was 0.74(0.21), indicating that DHL knowledge instrument was moderately easy. Internal consistency of the instrument was good, with Cronbach's α = 0.791. The test-retest scores showed no significant difference for 26 out of the 28 items, indicating that the questionnaire has achieved stable reliability. The overall mean(SD) knowledge scores was significantly different between the patient and professional groups [74.35(14.88) versus 93.84(6.47), *p *< 0.001]. This means that the DHL knowledge instrument could differentiate the knowledge levels of participants. The DHL knowledge instrument shows similar psychometric properties as other validated questionnaires.

**Conclusions:**

The DHL knowledge instrument shows good promise to be adopted as an instrument for assessing diabetic patients' knowledge concerning their disease conditions and medications in Malaysia.

## Background

Diabetes and its complications pose a major health-care burden worldwide and present major challenges to patients, health-care systems, and national economies. The World Health Organization estimates that between 2000 and 2030, the world population will increase by 37% and the number of people with diabetes will increase by 114% [1]. Asia will be the major site of a rapidly emerging diabetes epidemic based on population growth, an increase in the elderly population (> 65 years of age), and the rate of urbanisation [1]. India and China will remain the two countries with the highest numbers of people with diabetes (79·4 million and 42·3 million, respectively) by 2030 [1]. In Malaysia, the prevalence of diabetes has escalated from 6.3% in 1986, to 8.3% in 1996, and to 14.9% in 2006 [2]. One in six Malaysians above the age of 30 years old has diabetes [2]. The dramatic increase in the prevalence of diabetes may be due primarily to the rise in obesity in Malaysia during the past decade, which has been found to be associated with rapid urbanization [2,3].

Research has shown that the provision of diabetes education by healthcare professionals on medications, diet, exercise, home glucose monitoring, foot care, and treatment modifications have improved clinical outcomes and the quality of life of patients [4-6]. Although knowledge alone does not guarantee a change in behaviour or lead to effective self-management [7], the assessment of diabetes-related knowledge is an important step towards providing individualize diabetes education programs and to evaluate the effectiveness of such interventions [8].

A literature search revealed that several instruments for assessing diabetes knowledge have been developed: the Michigan Diabetes Knowledge Tool (MDKT) [9], the Diabetic Knowledge Questionnaire (DKQ) [10,11], the Diabetes Knowledge Assessment (DKN) scale [12], the Revised Diabetes Knowledge Scale [13], the PedCarbQuiz (PCQ) [14] and the Diabetic Numeracy Test (DNT) [15]. Most of these instruments were developed and validated in the United States [9-11,14,15], whilst others were developed and validated in Australia [12] and the United Kingdom [13]. The earliest instrument, the MDKT was developed and validated in the 1970s [16]. Most of these instruments were developed in the English Language [9-11,14,15]. However, several instruments have since been translated to other languages such as Spanish [10,11], Portuguese [17] and Bahasa Malaysia [18,19].

These instruments varied widely in their assessment objectives. The MDKT assessed knowledge on general issues and insulin use [9], the DKQ assessed general diabetes knowledge [10,11], the PCQ assessed carbohydrate food recognition, carbohydrate food counting and the incorporation of carbohydrate counting in calculating insulin dose [14], whilst the DNT measured numeracy skills for diabetes (food label interpretation, calculation of insulin dose based on blood, glucose and carbohydrate corrections) [15].

The population in which these instruments were assessed varied between studies. Instruments were validated in adults with type 1 or type 2 diabetes [9,15], in adults with type 2 diabetes only [10,11], or in adolescents or children with type 1 diabetes [14,15].

Patient's knowledge on diabetes, hypertension and hyperlipidaemia and its medications can be used as one of the outcome measures to assess the effectiveness of educational intervention. In Malaysia, two instruments: the Orang Asli-Diabetes Knowledge Questionnaire (OA-DKQ) [18] and the MDKT [19] have been translated into Bahasa Malaysia and validated. The OA-DKQ assesses diabetes-related knowledge whilst the MDKT assesses general diabetes knowledge and insulin use. However to date, no instrument that assesses knowledge on other cardiovascular metabolic risk factors such as hypertension and hyperlipidaemia, in addition to diabetes knowledge has been developed and validated in Malaysia. Therefore, the aim of this study was to develop and validate a comprehensive questionnaire to assess patient's knowledge on diabetes, medications, and other cardiovascular metabolic risk factors which included hypertension and hyperlipidaemia, for use by Malaysians with type 2 diabetes mellitus.

## Methods

### Development of the Diabetes, Hypertension and Hyperlipidemia (DHL) knowledge instrument

The DHL knowledge instrument was developed for the community-based Cardiovascular Risk Factors Intervention Strategies (CORFIS) trial. The aim of this trial was to assess the efficacy of a chronic disease management strategy in the management of patients with diabetes and/or hypertension and/or hyperlipidaemia in Malaysia. This knowledge instrument was then used to measure the levels of diabetes-related knowledge of participants in the CORFIS trial. The face and content validity of the DHL knowledge instrument was established by a group of 12 experienced pharmacists and researchers, who went through three drafts of the instrument before producing the final version. The final draft was then piloted on 20 practising community and hospital pharmacists as well as on five diabetic patients in a tertiary hospital, to obtain their feedback concerning the clarity and relevance of the instrument. The DHL knowledge instrument was developed in the English Language as English is understood by a majority of Malaysians. It consists of 28 questions and 5 domains, with each item having true, false and don't know options. The 5 domains are: diabetes (5 items), hypertension (5 items), hyperlipidemia (5 items), medications (8 items) and general issues (5 items).

### Patient Group

Patients with type 2 diabetes, currently on medications for their diabetes and who could communicate in English were included in the study. Excluded were children (< 18 years of age) and those who were not taking any medication for diabetes. Patients answered the DHL knowledge instrument twice: at baseline and 4 weeks later.

### Professional Group

To obtain a more objective assessment on the validity of the DHL knowledge instrument, pharmacists were recruited (from a tertiary hospital) as the professional group. Pharmacists were expected to have higher knowledge of diabetes than patients. The pharmacists answered the DHL knowledge instrument once only.

### Procedure

Patients were recruited from the diabetes clinic of a tertiary hospital in Kuala Lumpur, Malaysia. Baseline information such as demographic data, medical history, life style and medication history were collected. The questionnaire was administered whilst the patient was waiting to see the doctor. Patients completed the questionnaire themselves, after instructions were given by the researcher. Participants took about 10-15 minutes to complete the DHL knowledge instrument. The completed questionnaire was checked by the researcher to ensure that all questions had been answered. The DHL knowledge instrument was administered again to the same group of patients after one month, during their follow up visit at the clinic. Patients were questioned on any significant changes or events that had occurred within the one month interval and all changes were documented. This study was approved by the Medical Ethics Committee of the hospital under study.

### Statistical analyses

All data were entered and analyzed using the Statistical Package for Social Sciences (SPSS) version 15. The answers for each question in the knowledge questionnaire was scored as true, false or don't know. Analyses was performed by scoring 1 for a correct response and 0 for an incorrect or don't know response. The total score was converted into percentage, ranging from 0 to 100 percent. Each domain score was also analyzed by summing the scores of correct answers and converted into a scale of 0 to 100. Zero indicates the lowest level of knowledge, whilst 100 indicates the highest level.

### Analysis of the individual (dichotomous) items

#### The McNemar's test

This test was used to examine the test-retest reliability on the individual items.

##### Flesch reading ease

The Flesch reading index is a tool for estimating the reading comprehension level necessary to understand a written document based on the average number of syllables per word and the average number of words per sentence. Scores range from 0 to 100 indicates the level of difficulty in understanding the document, with lower numbers indicating greater difficulty. An average document should have a score of 60-70 [20].

##### Difficulty factor

The difficulty factor is defined by the proportion of patients selecting the right answer to that question and is calculated by the number of correct response divided by the total number of responses. It is a measure of how difficult the question was. The higher the difficulty factor, the easier the question is. A value of higher than 0.75 is deemed to be poor as the question may be too easy. Items with difficulty values between 0.3 and 0.7 are most effective. The optimal level should be 0.5 [21].

##### Cronbach's α

The internal consistency of the DHL knowledge instrument was assessed using Cronbach's α coefficient. Cronbach's α value ≥ 0.70 is considered as having good internal consistency. If omitting an item increases Cronbach's α significantly, then excluding the item will increase the homogeneity of the scale [22].

##### Analysis of the different domain scores

The mean knowledge score [standard deviation (SD)] was calculated for each domain. This score reflects the level of knowledge of each domain. Since the data obtained were not normally distributed, non-parametric tests were used.

The Wilcoxon Signed-rank test was used to compare if there was any significant difference between the test and retest results. The Mann-Whitney *U *test was used to compare if there was any significant difference between the patient and the professional group.

Spearman's rho was used to determine correlation coefficient of the domain scores of the patient group at test-retest.

##### Factors associated with knowledge scores

Spearman's rho and Chi square tests were used to determine if there were any relationship between demographic factors and knowledge scores for continuous and categorical variables, respectively. A p value of < 0.05 was considered as statistically significant.

## Results

A total of 117 participants were recruited: 77 (65.8%) patients and 40 (34.2%) pharmacists (professionals). Of the 77 patients, 37 (48.1%) were female. Sixteen patients (15.5%) were excluded in the test-retest analysis as they did not come for the second follow-up visit even after being reminded through telephone calls, leaving a total of 61 patients. Demographic characteristics of patients are as shown in Table 1.

**Table 1:** Patients' demographic characteristics

		No. of patients (n = 77)
Gender [n (%)]		

	Female	37 (48.1)

	Male	40 (51.9)

Mean age ± SD (years) [range]		62.53 ± 10.97 [25-82]

Ethnicity [n (%)]		

	Malay	11 (14.3)

	Chinese	21 (27.3)

	Indian	45 (58.4)

Mean BMI ± SD (kg/m2) [range]**		27.17 ± 4.69 [17.7-51.9]

BMI range [n (%)]**		

	< 18.5 (underweight)	1 (1.4)

	18.5-24.9 (normal)	21 (30.0)

	25.0-29.9 (overweight)	33 (47.1)

	>/= 30 (obese)	15 (21.4)

Level of education		

	≤ 6 years of education (none or primary)	3(3.9)

	12 years of education (secondary)	38 (49.4)

	≥ 15 years of education (diploma/tertiary)	36 (46.8)

Monthly income (RM) [n (%)]		

	None	58 (75.3)

	< 1000	2 (2.6)

	1000-3000	9 (11.7)

	3001-5000	6 (7.8)

	5001-10,000	2 (2.6)

Currently employed [n (%)]		19 (24.7)

Mean duration of diabetes ± SD (years) [range]		19.72 ± 9.98 [3-47]

Exercising ≥ 3 times per week [n (%)]		49 (64.5)

SD = standard deviation; BMI = body mass index; RM = ringgit Malaysia

**Data unavailable for 7 patients

### Psychometric properties of the DHL knowledge instrument

Flesch reading ease was 60, which was considered as satisfactory. Internal consistency for the overall DHL instrument was excellent (Cronbach's α = 0.791).

Corrected item-total correlations were first used to identify items which did not agree well with the other items. Item-total correlations should exceed 0.2 to be considered as acceptable [1]. Using this criterion, items 5, 7, 9, 17, 19 and 28 did not meet this requirement (Table 2). The Cronbach's α value for each item that represented the effect of removing that item from the calculation of the overall Cronbach's α value was also computed. The results showed that the internal consistency of the DHL knowledge instrument remained close to the overall Cronbach's α of 0.791 with the removal of any of these items. Therefore, all 28 items were retained. The difficulty factor for most of the items was satisfactory (0.14-0.92) with a mean(SD) of 0.74(0.21). Out of 28 questions, 8 had a difficulty factor of less than 0.75. This indicates that the knowledge instrument was moderately easy.

**Table 2:** The psychometric properties of the DHL instrument

			Difficulty factor	Corrected Item-total correlation	Cronbach's α if item is deleted	Test retest reliability
Domain	Item					*p*-valuet‡
	1.	Diabetes occurs in people with insufficient or no insulin#	0.81	0.406	0.780	0.508
	
	2.	Diabetes can be cured after taking medicines for a period of time	0.79	0.458	0.777	0.791
	
	3.	As long as a diabetic person's fasting blood sugar level in the person's fasti morning is in the normal range, he/she can eat anything for that day	0.88	0.377	0.782	0.453
	
	4.	If the blood sugar level is high for long period of time, it may cause other health problems such as blindness#	0.92	0.340	0.784	1.000
	
Diabetes	5.	Normal fasting blood sugar is between 4 to 6 mmol/L#	0.90	0.128	0.791	0.453

	6.	There is no problem for our blood pressure to remain high as long as we do not feel sick	0.73	0.548	0.771	0.125
	
	7.	Blood pressure of 140/90 mmHg and above is considered as high#	0.78	0.190	0.790	0.791
	
	8.	If not treated, high blood pressure can lead to kidney damage#	0.70	0.378	0.781	0.180
	
	9.	We can feel whether our blood pressure is high or not	0.23	0.125	0.794	0.189
	
Hypertension	10.	High blood pressure can be caused by hardened or narrowed blood vessels due to fatty deposits#	0.78	0.270	0.786	1.000

	11.	LDL cholesterol is known as "good" cholesterol	0.14	0.209	0.789	1,000
	
	12.	High level of "bad" cholesterol blocks blood vessels the risk of a heart attack#	0.86	0.252	0.787	0.453
	
Hyperlipidaemia	13.	High level of "bad" cholesterol" can also occur in thin people#	0.86	0.381	0.781	1.000
	
	14.	Cholesterol is present in some food and also produced in our liver#	0.69	0.267	0.787	0.238
	
	15.	Omega-3 supplements can reduce "bad" cholesterol more than the medicine given by the doctor	0.29	0.377	0.781	0.019*

	16.	We can stop taking medicine(s) once our blood sugar/blood pressure is well controlled.	0.81	0.450	0.777	0.109
	
	17.	All medicines must be taken after meals only	0.74	0.179	0.791	0.508
	
	18.	If someone misses taking his/her medicine, he/she can take double the amount for the next dose	0.92	0.518	0.779	0.375
	
	19.	People with type 1 diabetes cannot depend on tablets or oral type of medicines to control their blood sugar#	0.42	0.157	0.794	0.011*
	
	20.	All medicines for diabetes are the same, so we can share them if we have diabetes	0.88	0.273	0.786	0.727
	
	21.	Medicines for diabetes or high blood pressure can be taken on alternate days to reduce side effects	0.84	0.383	0.781	1.000
	
	22.	Medicine for reducing cholesterol only has to be taken just before taking any oily or fatty foods	0.83	0.354	0.782	1.000
	
Medication	23.	All medicines must be stored in the refrigerator	0.90	0.396	0.781	0.388

	24.	Tobacco smoking increases risk of heart diseases#	0.90	0.385	0.782	0.125
	
	25.	If you do not take any white sugar, you will not have enough energy	0.81	0.355	0.782	0.549
	
	26.	Vegetable oils do not contain cholesterol, therefore they are safe to be taken in large amount	0.75	0.311	0.784	1.000
	
	27.	30 minutes of exercise per week is enough to reduce the risk of getting heart problems	0.78	0.233	0.788	0.424
	
General Issues	28.	Diabetic people can eat as much fruits (such as banana, papaya, orange, water melon) as they like	0.90	0.065	0.794	0.062

#Statements are true

‡Test-retest reliability on the individual (dichotomous) items was conducted using the McNemar's test (binomial distribution was used)

*Statistically significant at *p *< 0.05

Test-retest reliability was assessed in 61 patients after a 4-week interval and all domains showed high correlation coefficients (0.391-0.559, *p *< 0.001). Out of 28 items, only two items (items no. 15 and 19) were found to be significantly different (Table 2). There was also no difference between the test-retest scores for all domains and the overall score (exceptions were the domains on hypertension and hyperlipidaemia) (Table 3).This indicates that the DHL knowledge instrument has achieved stable reliability.

**Table 3:** Knowledge scores of the professional and patient group at test and retest (by domain)

	Test	Retest	Test-retest reliability [A] versus [B]			Discriminant validity [A] versus [E]
	**Patients (n = 77)** **[A]**	**Patients** **(n = 61)#** **[B]**	**Wilcoxon Signed Rank test** **[C]**	**Spearman's rho** **Correlation** Coefficient** [D]	**Professionals (n = 40)** **[E]**	**Mann Whitney U test** **[F]**

Domain	Mean ± SD	Mean ± SD	*p-value*		Mean ± SD	*p-value*

Diabetes	85.97 ± 19.21	91.15 ± 15.29	0.318	0.391	95.50 ± 9.59	0.002*

Hypertension	64.41 ± 23.76	63.61 ± 20.50	0.018*	0.490	92.50 ± 9.81	< 0.001*

Hyperlipidaemia	56.62 ± 23.26	85.97 ± 19.21	0.018*	0.460	93.50 ± 11.45	< 0.001*

Medication	79.22 ± 20.14	79.51 ± 17.08	0.359	0.552	97.19 ± 5.29	< 0.001*

General issues	82.60 ± 20.09	81.97 ± 19.90	0.226	0.559	88.50 ± 17.48	0.100

Total knowledge score	74.35 ± 14.88	78.04 ± 12.09	0.215	0.617	93.84 ± 6.47	< 0.001*

#16 patients were excluded from the test-retest as they did not come for their follow-up visit

*Statistically significant at *p *< 0.05

**All correlations were statistically significant at *p *< 0.01

### Baseline diabetes-related knowledge levels of patients and professionals

Table 3 shows the baseline knowledge scores for the patient and the professional group. The mean scores for diabetes, hypertension, hyperlipidaemia and medication were significantly lower in the patient group compared to the professional group, which then affected the overall mean(SD) knowledge score [74.4(14.9) versus 93.8(6.5), *p *< 0.001] (Table 3). Patients had fairly good knowledge on diabetes and its general issues, followed by knowledge on medications, hypertension and hyperlipidemia. The higher the education level of the patient, the better their knowledge score (Spearman's rho = 0.377, *p *= 0.001). Other demographic factors such as gender, age, BMI, ethnicity, monthly income, employment status, duration of diabetes and exercise, were not associated with knowledge scores.

### Comparison of the DHL knowledge instrument with other validated instruments

The DHL instrument was compared with other validated instruments for assessing knowledge on diabetes (Table 4). The psychometric properties of the DHL knowledge instrument were similar to these other validated instruments. For example, the Flesch reading ease (or equivalent) was around 60 and Cronbach's alpha value was above 0.7.

**Table 4:** Comparison of psychometric properties of the DHL instrument with other validated instruments for assessing knowledge in diabetic patients

	DHL	MDKT	DKQ24#	PCQ	DNT
Mean age [range] (years)	62.53 [25-82]	60.00 (community participants) 56.00 (health dept participants)	50.27 [20-79]	13.2	54.2

No. of subjects	77	811	502	75	398

No. of items	28	23	24	78	43

No. of domains	5	2	1@	7	5

Flesch reading ease (Flesch Kincaid grade level)	60	(6th grade)	NR	(6)	NR

Cronbach's α or Kuder Richardson (KR)	0.791	0.710	0.78	0.88	(0.95)

Difficulty factor	0.74	NR	0.57	NR	NR

Mean score (%)	74.35	54.02 (general use) 44.44 (insulin use)	57.00	87.00	61.00

DHL = Diabetes, hypertension and hyperlipidaemia knowledge instrument

MDKT = Michigan Diabetic knowledge tool [9]

DKQ60 = 60 item diabetic knowledge questionnaire [11]

DKQ24 = 24 item diabetic knowledge questionnaire [10]

PCQ = PedCarbQuiz [14]

DNT = Diabetic numeracy test [15]. A more clinically useful 15 items DNT is available

#All 60 items of the DKQ were administered at all data collections; only data pertaining to the 24 items were used for analysis

NR = not reported

@Instrument not divided into domains

## Discussion

The DHL developed in this study performed satisfactorily for most of the psychometric components analysed.

The DHL knowledge instrument had a Flesch reading ease of 60. This is within the preferred range score of 60-70, indicating that this instrument is suitable for the average adult to read. Other validated instruments like the DKT [9] and the PCQ [14] were found to have a 6th Flesch Kincaid grade level, which indicates that the text is expected to be understandable by an average student in the 6th grade (usually around ages 11-12 in the United States of America). The overall Cronbach's α was good (0.791).

The difficulty factor for most of the items was satisfactory (0.14-0.92) with a mean(SD) of 0.74(0.21). Out of 28 questions, 8 had a difficulty factor of less than 0.75. This indicates that the knowledge instrument was easy for patients to answer, which was further substantiated by the mean baseline knowledge score of 74.4(14.9)%. Other studies on the validation of instruments that assessed diabetes-related knowledge obtained lower scores which ranged from 44.4-61.0% [9,10,15]. The only exception was the study by Koontz et al. [14] which obtained a score of 87.0%. Patients in the present study showed the highest knowledge on diabetes [86.0(19.2)%], and least knowledge on hyperlipidemia [56.6(23.3)%]. This finding is not unusual as not all diabetic patients have hyperlipidemia or hypertension and hence they may not have been provided with information on these conditions. However, these three diseases are inter-related, and patients should know about the cardiovascular risk factors associated with diabetes.

The reliability of the DHL was excellent as shown by the test-retest results. Out of 28 items, only 2 items showed a significant difference in the test-retest results which affected the domain scores on hypertension and hyperlipidaemia. Item 15 ("Omega-3 supplements can reduce "bad" cholesterol more than the medicine given by the doctor") showed a significant improvement in the retest, indicating that patients may have checked the information after the initial test. As for item 19 ("People with type 1 diabetes cannot depend on tablets or oral type of medicines to control their blood sugar"), less patients answered these statements correctly at retest. A possible reason is that the patients "guessed" the answers correctly during the initial test, but could not remember their previous answer and hence "guessed" wrongly the second time.

The overall knowledge scores of the DHL instrument were higher in the professional group (pharmacists) than in the patient group (93.8% versus 74.4%) as well as in all domains. Pharmacists are expected to have higher level of knowledge on diabetes and its related issues compared to patients. This indicates that the DHL instrument is a valid instrument that can be used to differentiate the knowledge levels of diabetes amongst its participants.

One of the limitations of this study was the small sample size. It has been recommended that the number of participants required should be the number of items (that is, 28 items) multiplied by 5-10. This means that at least 140 participants are required. In addition, the DHL knowledge instrument was only evaluated in the English language but Malaysia is a multi-racial country. Therefore, results of this study may not be applicable to patients who cannot read or understand English. Further studies using the Bahasa Malaysia and Mandarin versions of the DHL knowledge instrument are required to enable the results to be more representative of diabetic patients in Malaysia.

## Conclusions

The psychometric analysis showed that the DHL knowledge instrument has a satisfactory difficulty factor and shows good promise to be adopted as an instrument for assessing patients' knowledge about diabetes and its medications as well as other cardiovascular metabolic risk factors in Malaysia. The DHL knowledge instrument can be used to identify individuals in need of educational interventions and to record changes over time as treatment progresses for each patient in Malaysia.

## Competing interests

The authors declare that they have no competing interests.

## Authors' contributions

PSML researched data, contributed to discussion and wrote the manuscript; SSC moulded the study, designed the questionnaire, contributed to discussion and reviewed/edited the manuscript; CHT collected and researched data, contributed to discussion and reviewed/edited the manuscript; SPC contributed to discussion and reviewed/edited the manuscript. All authors read and approved the final manuscript.

## Pre-publication history

The pre-publication history for this paper can be accessed here:

http://www.biomedcentral.com/1471-2288/12/18/prepub
